# Process evaluation of podiatric treatment of patients with forefoot pain

**DOI:** 10.1186/1757-1146-6-32

**Published:** 2013-08-07

**Authors:** Babette C van der Zwaard, Wim JC Swagerman, Benedicte Vanwanseele, Kees J Gorter, Henriëtte E van der Horst, Petra JM Elders

**Affiliations:** 1 EMGO+ Institute, Department of general practice and elderly care medicine, VU University Medical Centre, Amsterdam, Netherlands; 2 Lectoraat, Fontys University for Applied Sciences, Eindhoven, Netherlands; 3 Department of Kinesiology, KU Leuven, Leuven, Belgium; 4 Department of General Practice, University Medical Centre Utrecht, Utrecht, Netherlands

**Keywords:** Forefoot pain, Podiatric treatment, Process evaluation, Aetiology, Footwear advice, Foot orthoses

## Abstract

**Background:**

Foot pain is a common problem for people aged 50 and over and occurs more often in women than in men. About 60% of the foot problems are forefoot problems and slightly more than half of these patients seek medical help, mainly in the form of podiatric care. Podiatric treatment of forefoot problems is known to be heterogeneous. The aims of the present study are to describe the podiatric treatment of patients with forefoot pain and to evaluate the podiatric examination and treatment using an expert panel.

**Method:**

We invited twenty-five randomly selected subjects with forefoot problems who had received podiatric treatment in a pragmatic randomised clinical trial to participate in an analysis of their treatment by an expert panel. The panel retrospectively established the cause of the foot problem as well as the therapeutic goals and evaluated the treatment. These findings were compared to those reported by the treating podiatrist.

**Results:**

Two fundamentally different approaches were found in approach of podiatric examination; a functional approach (n =13) and a non-functional approach (n =12). In nine cases the expert panel agreed with the cause recorded by the podiatrist. In five other cases the expert panel concluded that the treatment of the podiatrist was not consistent with the cause of the problem recorded by the podiatrist. Of the 10 patients for whom the podiatrist had recorded to have given shoe advice, only two were able to recollect the proper advice. Three patients did not remember receiving advice at all.

**Conclusion:**

In this study almost half of the podiatrists worked according to a non-functional approach where the other half (like the expert panel) chose a functional strategy that analyses the underlying problem. Fundamental differences in treatment plans and thus heterogeneous treatments could be a consequence.

## Background

Foot pain and subsequent functional limitations are common in aging people. Prevalence figures between 15% and 42% have been reported in people over 50 [1-6]. Foot pain occurs more often in women than in men [1,2] and the forefoot is affected more often than any other part of the foot [1,2,7]. In some cases foot pain is known to lead to decreased mobility [2,5,8-10], increased risk of falling [6,11] and a lower experience of well-being [2,8].

According to a Dutch survey, 56% of people with foot complaints or pain sought medical help, mostly with a podiatrist (46%) or with a general practitioner (GP) (36%) [7]. Dutch GPs treat foot problems by prescribing NSAIDs, providing life-style advice (e.g. lose weight, wear other shoes), referring to a paramedical professional or referring to a medical specialist [12]. When referred, most patients are referred to a podiatrist [12]. Podiatric treatment in the Netherlands may consist of skin and nail care, providing information on footwear or providing an insole or foot orthotics [13].

Podiatric treatment is heterogeneous between different countries because the treatment can be based on theoretical concepts like that of Root *et al*. [14,15] or the concept of Lavigne *et al*. [16]. Both concepts are similar in the approach in which foot pain is related to an anatomical or kinematic impediment and in which treatment is aimed at correcting or reducing the effect of the underlying impediment (i.e. analysing the kinetic chain). The main difference between these concepts is the way the orthotic is fabricated. Besides these possible differences, even within countries in which a same treatment concept is used patients receive different treatments for similar problems [13,17-19] and inter-practitioner variability is known to exist [19]. Most of the podiatrists in the Netherlands have received their formal education at the Fontys University of Applied Sciences whose curriculum is mainly based on the concept of Lavigne. The heterogeneity of the treatments could partly be due to the lack of reliability of important components that are part of the physical examination [20].

Both the treatment and physical examination are components of the entire treatment process. The procedures and routines of podiatrists and the choices they make during the different stages of diagnosis and treatment are currently unidentified. The aims of the present study are: (i) to describe general podiatric treatment of patients with forefoot pain and (ii) to evaluate the entire treatment process using an expert panel.

## Method

### Participants

Twenty-five patients who had been treated by a podiatrist for forefoot pain as part as a larger intervention study [21] were randomly selected and invited to participate in this process-analysis of podiatric treatment. These participants had visited their GP with a functional impeding forefoot pain between March 2010 and May 2012 and were randomly allocated to be treated by a podiatrist. The medical ethics committee of the Vrije Universiteit medical centre has approved the study (2009/267).

### Podiatric treatment

**Figure 1:**
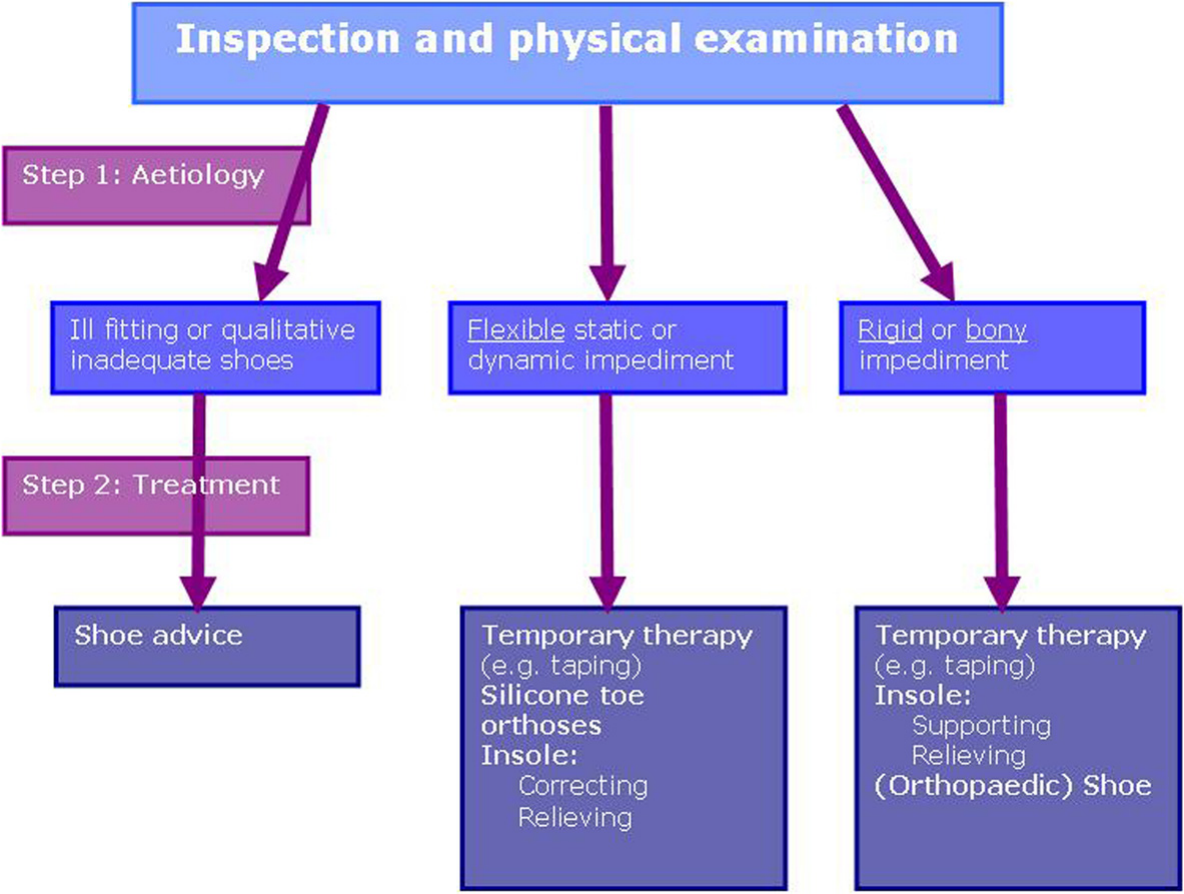
**Podiatric diagnostic and treatment framework.** The podiatric diagnostic and treatment framework was developed in cooperation with the Fontys University of Applied Sciences and five podiatrists.

Thirteen podiatrists provided the podiatric treatment. All podiatrists received instructions about the treatment framework (Figure 1). They filled in a standardised form with the aetiology of the foot problem, the aim of their treatment and the content of the delivered treatment immediately after they had completed their treatment. If treatment by means of orthotic devices was chosen the specific elements of the orthotic device was to be recorded by the podiatrist.

### Diagnostic and therapeutic framework

Evidence on treating forefoot problems is currently lacking. A method was needed for this trial that enabled us to describe the podiatric treatment process in a standardised manner without losing the ability to incorporate individual patient-driven attributes. Together with Fontys University of Applied Sciences a podiatric diagnostic and treatment framework was developed (Figure 1). We asked five podiatrists to use the framework and provide suggestions for improvement. All of them indicated that they could work with the framework and did not have any suggestions for changes.

### Expert panel

In order to evaluate the entire podiatric treatment process an expert panel was formed. The panel consisted of three experts: two lecturers and an orthopaedic surgeon. Both lecturers are affiliated with the podiatry course of the Fontys University of Applied sciences. The first lecturer is a practising podiatrist and the second lecturer is a human movement scientist specialised in anatomy, biomechanics and gait analysis. We asked the orthopaedic surgeon to state if he would have proposed an operation if he saw the patients in his office. A practising GP was asked alongside the expert panel to provide information regarding the GP perspective on treatment choices.

### Data collection

As previously stated, the participants in this study were randomly selected from a pragmatic RCT [21]. During the inclusion for this RCT, fourth year podiatry students performed several examinations on all patients as part of their final project supervised by the lead researcher (BvdZ). These examinations consisted of the following elements: patient-reported area of foot problem on a foot manikin [1]; diabetic neuropathy screening [21]; photographs of the medial, lateral and dorsum of both feet and shoes [21]; the Foot Posture Index [22,23]; an evaluation of the shoes (function and fit); barefoot pressure during gait [24,25]. These examinations were prospectively established to facilitate the process evaluation of the podiatric treatment and were made available to the expert panel together with information about age, gender, weight and height of the subjects, and the completed treatment form by the treating podiatrist.

The expert panel also collected data. One of the lecturers from the expert panel carried out a physical examination of every participant in this trial while the other lecturer was observing. Both performed a visual gait analysis. A ‘thinking out loud’ method was used during all examinations and everything was recorded. Consensus between the members of the expert panel was reached during the examinations. Examinations were repeated if needed to reach a consensus. The number of discussions and re-examinations was scored by evaluating the recordings afterwards. All participants were interviewed by BvdZ to establish if the situation of the foot pain had been altered since the commencement of the treatment. If changes had occurred, the expert panel attempted to acquire information from the patient about the foot pain preceding the treatment. The expert team used all the collected data to reconstruct the situation comparable to the one prior to podiatric treatment.

Interviews established whether patients remembered and understood the information provided by the podiatrists.

### Expert team evaluation procedure

First, the expert panel reviewed the data collected during the inclusion measurements then carried out the interviews and physical examinations. Next, the expert panel evaluated the podiatric treatment reported by the podiatrist. A total of eight sessions were needed to evaluate all patients. The orthopaedic surgeon did not physically attend these sessions but provided his opinion prospectively and retrospectively both in person and via e-mail to one of the lecturers. The same lecturer met with the GP on three occasions.

The evaluation consisted of three elements: first, the possible cause of the foot problem(s) and the corresponding therapeutic goal(s) reported by the podiatrist were evaluated by comparing them to those established by the expert panel (element 1). The probable cause of the foot problem and the subsequent treatment goals and therapeutic choices were discussed within the expert panel until consensus was reached. Both the GP as well as the orthopaedic surgeon added information about the treatment choices they would make when treating the patient. Secondly, the expert panel evaluated the therapeutic consistency of the podiatrist using the diagnostic and therapeutic framework (Figure 1) until consensus was reached (element 2). The third and last element consisted of using the information derived from the interviews. The recollection and comprehension of information by the patient was compared to the provided information as reported by the podiatrist (element 3).

## Results

### Study population

We contacted thirty-eight randomly selected participants who had been seen by the podiatrist. Thirteen patients declined to participate in this part of the study. This was due to health related causes in four cases; nine patients could not come on the days of the examination or were too busy in that period. Finally, twenty-five participants aged 66 years (SD 9.1) were examined by the expert panel. Examinations took place on average three months (SD 2.4) after the first visit to the podiatrist. In 56,5% of the cases, symptoms had been experienced for more than two years, while in 21,7%,between one and two years. Patient characteristics are shown in Table 1. The two most affected locations of the foot were the plantar side underneath metatarsophalangeal joint 1 (52%) and 2–5 (48%). Bilateral pain was reported by 61% of the participants.

**Table 1:** Patient characteristics

	**Age**	**Period since**	**Duration of the**				
	**(Years)**	**treatment**	**symptoms (Months)**				
		**(Months)**					
	**Mean (SD)**	**Mean (SD)**	**1-3**	**3-6**	**6-12**	**12-24**	**>24**
**Male (n = 4)**	69 (4)	3 (2,3)	0	0	0	1	3
**Female (n = 21)**	64 (10)	3 (2,4)	2	1	3	4	11
**Total (n = 25)**	65 (9)	3 (2,4)	2	1	3	5	14

A total of thirteen podiatrists treated the twenty-five patients. Two podiatrists (five patients) neglected to return the framework form. In these cases the expert panel tried to derive the cause and therapeutic goal of the podiatrist by analysing the fabricated insole. This was deemed possible because in all these cases an insole-element was used that solely redistributes and divides the plantar pressure underneath the MTP joints. These five patients were excluded for therapeutic consistency (element 2) or information comprehension of the evaluation (element 3).

### Expert panel evaluation process

In seven cases, after the evaluation of the data of the inclusion examination, the members of the expert panel did not agree on the possible cause of the symptoms. All possible causes were recorded and explicitly discussed during the ensuing physical examination using a ‘thinking out loud’ method. This consisted of the following: one lecturer (with the practical podiatrist background) performed the physical examination and expressed findings and conclusions, while the second lecturer was looking on and agreed or disagreed. For every disagreement, the examination was repeated and discussed until consensus was reached. A total of forty-one disagreements were solved in this way; ranging from zero to five per participant. All discrepancies were solved and consensus was reached for every participant.

The orthopaedic surgeon’s primary policy would normally be to have an X-ray made for all patients prior to diagnosis. Based on the photographs (and depending on the expected results of the X-rays) this surgeon would have considered operating in eight patients’ cases. These were mainly cases with Hallux Valgus deviations. The general practitioner evaluated the patients’ diagnoses established by the expert panel and also gave an opinion on the treatment options available for a GP. The array of treatment choices according to the expert panel, but also the GP and orthopaedic surgeon is shown in Table 2.

**Table 2:** Summary of results

**ID**	**Cause**^**I**^**:**	**Agreement PT and**	**Chosen therapy:**		**Shoe advice**		**Lifestyle advice**	**Foot pain at time of EP**	**Information**	**Chosen**	**Chosen**	**Consistency cause**
		**EP on cause**	**insole elements**^**II**^		**regarding**^**III**^**:**			**examination compared**	**comprehension**	**therapy**^**IV**^**: GP**	**therapy**^**IV**^**:OS**	**and treatment PT**
								**to time of inclusion**	**and recollection**			**according to EP**
									**shoe advice**			
			**PT**	**EP**	**Fit**	**Quality**						
**1**	TPM (NF)	No	d, e	d	EP			Less pain	TPM	d	c, possibly f	TPM
**2**	NF	No	a, b	g, (a)	EP			More pain	None provided	d	d	Yes
**3**	F	Partial	a, b, c, f	(a)		PT		Unchanged	Partial compreh.	g, h	c, f	Yes
**4**	NF	No	a, b			EP*		Less pain	None provided	f	f, i	Yes
**5**	TPM (NF)	No			EP*			Unchanged	TPM	g, d, possibly b	f, possibly k	TPM
**6**	NF	No	a, b, e		EP*			Less pain	None provided	f, d, g	j	Yes
**7**	NF	No	a, b, g		EP*			Less pain	None provided	f, d	f	Yes
**8**	TPM (NF)	No	a, c, g		EP*	EP		Less pain	TPM	f or possibly h	f	TPM
**9**	F	Yes	a, b, d, e	b		PT, EP		Less pain	Comprehended	d, h	k	Yes
**10**	F	Yes	a, b, d	a, b, c	PT, EP			More pain	No recollection	f, d, discuss: h	k	No
**11**	F	Yes	a, b, c	a, b				Less pain	None provided	f	k	Yes
**12**	F	Yes	a, b		PT, EP*	PT, EP		Less pain	Comprehended	f, b	f, possibly k	Yes
**13**	F	Yes		a, g		PT, EP		Unchanged	Comprehended	d, if persisting: f	f, i	Yes
**14**	F	No	a, b, e			PT, EP		Unchanged	Partial compreh.	f, possibly h	k	Yes
**15**	NF	No	a, b	a, d	EP*	PT, EP	EP (exercises, weight red.)	More pain	No recollection	g, d, e (dietary)	k, c	No
**16**	NF	No	a, b, e		EP	EP		More pain	None provided	f (foot), h (hip)	X-ray hip and go from there	No
**17**	NF	No	a, b, c, g	e	EP*	PT, EP	EP (exercises, weight red.)	More pain	Partial compreh.	f, d	c, f	No
**18**	TPM (F)	Partial	b,g	a	EP			Less pain	TPM	h	f, possibly i	TPM
**19**	TPM (NF)	No	a, b, c, d		EP*			Unchanged	TPM	f	f, possibly k	TPM
**20**	F	No	a, b, c, e		EP*		EP (basic foot care)	Less pain	None provided	f	f	Yes
**21**	F	No	a, b	g	EP	PT		Unchanged	Partial compreh.	d, f, e (dietary)	f	Yes
**22**	F	Yes	b, c, d		PT*, EP*	PT, EP		Less pain	Partial compreh.	a, g, f	f possibly k	Yes
**23**	NF	No	a, b, c, d	a, b	EP			More pain	None provided	f, d	f	Yes
**24**	F	Yes	a, b, c	a, b, c	PT, EP	PT, EP		Less pain	Comprehended	f, possibly g	f	Yes
**25**	F	Yes	a, b, c, f		PT*, EP*	PT, EP		Less pain	Comprehended	d, f possibly g	f	Yes

Podiatrists (PT) reported their results following their assessment of the participant on a standardised form and the Expert Panel (EP) discussed and reported their results after evaluating all information and performing a physical examination.

^I^*NF* Non-Functional strategy, *F* Functional strategy, *TPM* Treatment Plan Missing: Did not receive a treatment plan from the PT.

^II^Dutch orthotic devices are generally custom made insoles using the ‘Lavigne’ method [16]. Elements are divided in: retro capital support (a); arch support (b); stabilisation of the calcaneus (c); element to raise single or multiple MTP joints (d); rear foot pronator (e); rear foot supinator (f); heel lift (g).

^III^Shoes primary cause of foot problem.

^IV^*GP* (General Practitioner), *OS* (orthopaedic surgeon): NSAID’s (a); salicylic (10%) salve (b); night splint (c); shoe advice (d); life-style advice (e); referral insole fabrication (f); steroid injection (g); referral orthopaedic surgeon (h); referral neurologist (i); referral back to GP (j);operation (type depending on X-ray) (k).

### Element 1: Cause and therapeutic goals

**Figure 2:**
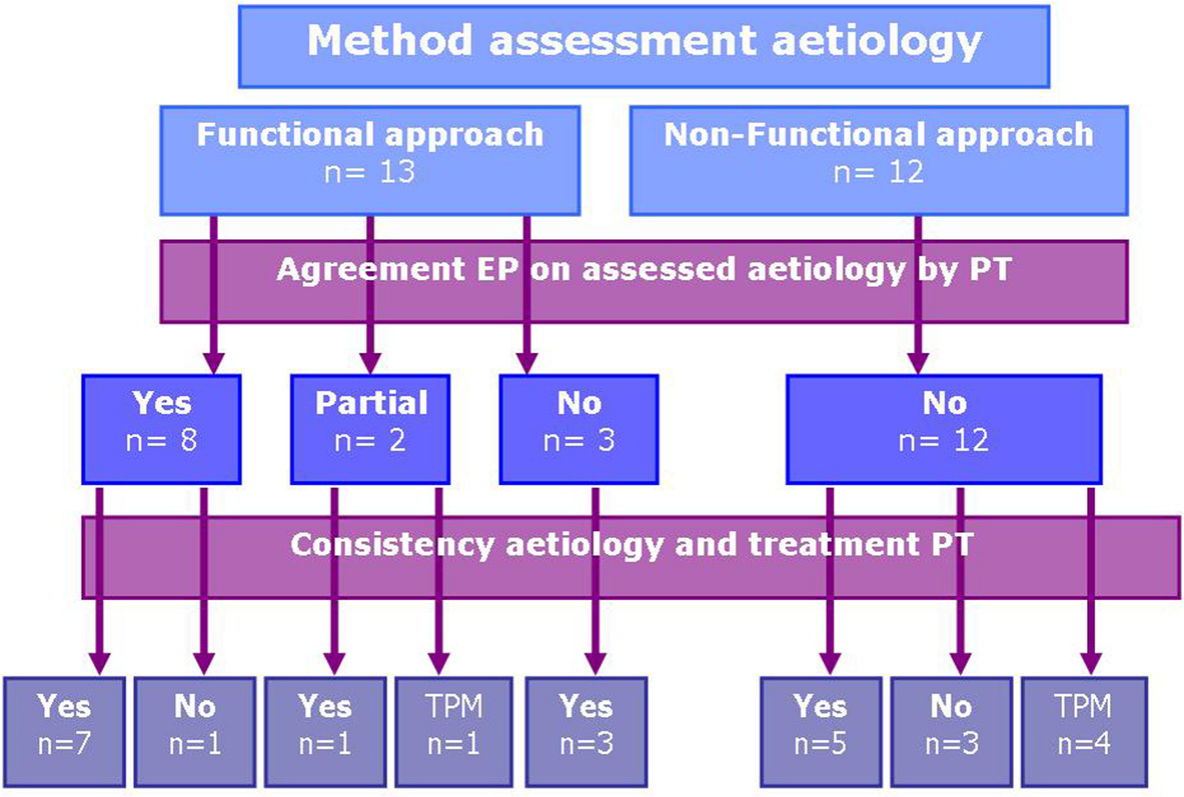
**Overview of different approaches to establish aetiology.** TPM= treatment plan missing.

Of the twenty-five causes established by the podiatrists, eight were considered to be correct by the expert panel (Table 2). After further analysis of the data it became evident that the approach of reaching a diagnosis differs between podiatrists. We saw two approaches. First we identified the functional approach that was consistent with the approach of the expert panel. This is an approach in which the kinetic chain is evaluated in order to find underlying (kinematic) impediment of the foot problem or when external influences like foot wear are evaluated as a possible cause of the problem. Secondly we identified another approach in which the podiatrist described local symptoms as a diagnosis without evaluating possible impediments beyond the area of the symptoms; a non-functional approach. Examples of these approaches are shown in Additional file 1. Differences in approach during the analysis of the cause of the foot pain led to establishing different therapeutic goals. A summary of these findings is shown in Figure 2. In twelve of the cases, the expert panel concluded that the podiatrist merely identified non-functional causes.

Wearing shoes a size too small was found to be the sole cause of the forefoot pain in five cases according to the expert panel. In another eight cases the size was evaluated to be part of the cause in addition to an anatomical or functional anomaly. In four of these thirteen cases, podiatrists reported to have found the shoes to be a size too small and thus of influence on the development and continuation of the forefoot pain. In five other cases the podiatrist described the shoes to be too unsupportive (n = 3), too old (n = 1) and too stiff (n =1). The expert panel agreed with only the latter to be an actual cause of the foot problem. According to the interviews, two podiatrists did not look at the shoes until the insoles were already fabricated.

“*When I came back for my insole*, *she examined the sole to make sure it fitted the shoe well*.” (*Female 71yr*)

### Element 2: Therapeutic consistency

In the second step of the diagnostic and therapeutic framework (Figure 1), podiatrists were asked to select a therapy based on the probable cause of the symptoms. The expert panel analysed whether the cause of the problem as recorded by the podiatrist was consistent with the chosen therapy. Two podiatrists (five patients) did not fill in the form with therapeutic goals therefore the expert panel could not evaluate the therapeutic consistency of these therapies. In four of the remaining twenty cases, the chosen therapy was not consistent with the stated cause. Sixteen therapies were in-line with the recorded cause of the symptoms, (Table 2) although in five of these sixteen patients the cause recorded by the podiatrist did not correspond with the cause found by the expert panel (Figure 2).

### Element 3: Information comprehension

The expert panel evaluated if information provided by the podiatrist was comprehended by the participants who were treated, on average, 3 months prior to the interview (Table 1). Twelve podiatrists reported that they provided shoe advice to the patients as they deemed inadequate shoes to be part of the cause of the problem. Two of these patients stated that they did not remember receiving any shoe advice, while five remembered only parts of the advice or interpreted the advice incorrectly. Only five patients were able to reproduce the entire advice (Table 2).

“*I received a piece of paper which contained all sorts of information*. *She told me that I had to wear other shoes and if I were to buy shoes I should get shoes with laces and not to buy loafers*.” (*Man 72yrs*; *remembers the entire advice*)

“*A bit higher heel was better than no heel at all*, *he told me*, *and it should be a bit close*-*fitting and supporting*, *that*'*s what I remember*.” (*Female 56yrs*; *remembers part of the advice*)

"*I had a pair of boots which looked quite elegant but were still comfortable*, *with a high heel*, *which would also be good*, *as long as the sole fitted the shoe*. *I brought my Archer* (*shoe brand* – *ed*.) *with me and this pair of Nike*-*air shoes*. *No*, *those were good*, *those shoes*." (*Female 61yrs*; *remembers the advice incorrectly*)

## Discussion

This study was carried out in order to describe current podiatric treatment of forefoot problems and to evaluate the treatment process. Our analyses show that the proceedings of the podiatrists to reach a diagnosis are heterogeneous: they used either a functional or a non-functional approach. Secondly, the expert panel found that in five out of twenty cases the chosen therapy was not adequate for the reported cause of the pain. And lastly, the podiatrists reported to have provided shoe advice more often than the patients remembered.

The treatment framework that was developed proved to be an effective way to analyse and evaluate the steps taken in podiatric treatment. Using the framework, two different approaches to assess the aetiology of a patient’s problem emerged from the data and the difference in approach led to different diagnoses. The functional approach is the way of working based on the concepts of Root [14,15] and Lavigne [16], previously defined as a search for the underlying (kinetic) cause of a symptom or looking at external factors like foot wear. Most scientific research to do with foot related problems is based on a functional approach [26-31]. This indicates that it is the preferred approach to establish aetiology, even though its validity has neither been studied nor the approach proven to be the best approach in the treatment of forefoot problems.

When different aetiologies are established due to differences in approach it is apparent that the therapeutic goals and treatments may differ. Literature shows that podiatric treatments are heterogeneous [13,17,18] and that the effects of the treatments are heterogeneous as well [19]. It is possible that the poor reliability of clinical assessments [20] as well as the different approaches we identified provide an explanation to these reported differences. The difference in the approach to analyse a forefoot problem that has emerged from our study is noteworthy. Even though the sample of twenty-five participants is small, our study showed that almost half of the podiatrists do not adhere to the concepts taught during their training. In future studies it is advisable to allocate or ascertain the chosen approach when reporting findings, but more importantly, prospective research should be carried out to definitively establish which approach is more efficacious in problem reduction.

It has been shown that foot problems like lesser toe deformities and hallux valgus could be related to wearing shoes that are too small or of inadequate quality [19,32]. A causal relationship however, has never been established. We found that the podiatrist mentioned footwear less often as a possible and/or partial cause of forefoot pain than the expert panel (15 vs. 23). This can once more be partly explained by the differences in approach. It appears that many podiatrists in our study did not look beyond the problem area of the symptoms, as required when evaluating shoes. In only four of the thirteen cases established by the expert panel did the podiatrist report to have found the shoes the patient wore prior to visiting the podiatrist, to be a size too small or lacking in appropriate quality and thus of influence on the development and continuation of the forefoot pain. Therefore most podiatrists in this study manufactured a podiatric insole instead of merely providing shoe advice. Another reason could be that the financial stimulant of manufacturing an insole is bigger than that of merely providing advice. Most Dutch health insurance companies provide a separate reimbursement for the inspection and physical examination of the patient and for the insoles. The fee for the latter is usually higher than for the physical examination and it could be possible that podiatrists prescribe a treatment by means of insoles more often than necessary. Lastly, the influence of the patient should not be underestimated. A patient with the expectation of receiving insoles could influence the podiatrist in providing insoles.

A marked difference in preferred treatment choices within the expert panel is the treatment choice of the orthopaedic surgeon compared to the rest of the panel for patients with a hallux valgus. The other members would mainly treat the hallux valgus conservatively with an orthotic and/or shoe advice, or a night splint. In contrast the orthopaedic surgeon would operate on seven of the twenty-five patients if an X-ray would confirm deviation of the first metatarsal. According to the orthopaedic surgeon in the expert panel, the majority of his patients have already tried most or all conservative treatment possibilities. However, GPs and podiatrists see patients prior to the point that they have tried all options and visit the orthopaedic surgeon. For the orthopaedic surgeon the most important criterion to operate is to reduce the amount of pain or to stop progression of the hallux valgus. The limited number of studies available has shown that in contrast to an operation an insole is incapable of correcting an already existing hallux valgus [27], however, related symptoms have been shown to decrease when treating with a custom manufactured insole [28].

According to the expert panel, in four cases the therapy chosen by the podiatrist was not adequate for the cause of the foot problem recorded by the podiatrist. In all of these four cases the reported aetiology on the form was in accordance with a functional approach, but the chosen treatment was symptom driven. Therewith the provided treatment (insole) did not provide the patient with a therapy that was aimed at correction of or compensation for the cause of the foot problem as established by the podiatrist, possibly rendering the treatment less effective. In the interviews, none of these patients indicated improvement of their complaints after the treatment.

The Gorter *et al*. [12] study on the management of common foot problems by GPs shows that life-style advice (e.g. wear better shoes, lose weight) was provided to patients alongside other treatments. Although there is no evidence that providing such life-style advice will actually help, some evidence shows that people with a higher fat-mass are at higher risk of developing foot problems [33].

In this study, the podiatrists reported to have provided the patient with shoe advice in ten cases. However, it became apparent from the interviews that not all patients remembered the information correctly (n = 5) or even remembered receiving any information at all (n = 3). This could be explained in several ways. It could be that the podiatrists don’t provide shoe advice as much as they report they do or the patients don’t remember receiving advice when it is provided. The latter is a known problem in health care and it is proposed that health care providers should be aware of this problem in order to communicate more effectively [34]. It is also possible that the average time of three months that elapsed between the podiatrist providing the advice and this study is responsible for a diminished information recollection by the patient. Some patients mentioned that they would have preferred to have some form of written advice in addition to the verbal communication. An information leaflet might be a recommendation. We also suggest that the provision of shoe and/or life-style advice should be done in a separate session, in order not to overwhelm patients with information.

Our process evaluation of podiatric treatment has to be interpreted in context of the strengths and limitations of the study. One might consider the focus on forefoot problems a limitation. The results seen in this study should therefore only be applied to patients with similar problems. On the other hand, this restriction to forefoot problems ensures a homogenous population could be viewed as a strength. The diversity in backgrounds of the expert panel is an asset to the study. This way the aetiology and treatment has been analysed from several medical angles. In contrast, the size of the expert panel, small for pragmatic reasons, is a limitation of the study. In light of the striking findings of this study we would recommend a replication of this study with a prospective design. Two members of the expert panel examined the patients and did not base their evaluation merely on the data provided. The method of examination could also be seen as a limiting factor. The members did not examine the patient separately but simultaneously and discussed differences of opinion during the examination. This could have influenced the objectivity of the members. Nonetheless, forty-one discussions were conducted showing that the panel members did state differences in opinion. Another limiting factor is the fact that out of twenty-five participants two podiatrists treating five patients did not return any form (even after two reminders). We deduced the cause found by the podiatrists for these five patients by analysing the elements used in the insole and these findings are less reliable than is desirable. The evaluation of the consistency between recorded cause and executed therapy was impossible for these participants, so that analysis is based on 20 participants. Furthermore, the expert panel was not blinded for the current status of the patients, which could have influenced the evaluation.

This study indicates that the approach to reach a conclusion on aetiology of forefoot pain is heterogeneous amongst podiatrists. It could also explain part of the variability found between podiatric treatments as mentioned in other studies. Half of the podiatrists followed a non-functional approach that was inconsistent with usual treatment concepts and inconsistent with the functional approach of the expert panel. Most insole or foot orthotic related studies follow a functional approach however the superiority of a functional approach over a non-functional approach has not yet been established. It is possible that some but not all foot problems fare well with a non-functional approach. It is advisable that future podiatric effect studies analyse the approach as an additional variable. Third, the orally provided advice could be more effective. We suggest extending the oral advice with a written/photo supplement to enhance understanding and adherence or provide advice in a separate session.

## Consent

Written informed consent was obtained from the patient for the publication of this report and any accompanying images.

## Competing interests

The authors declare that they have no competing interests.

## Authors’ contribution

BvdZ was responsible for data-collection and wrote the manuscript, together with PE. WS assisted during data-collection and created the tables and Additional file 1. HH commented on several drafts of the manuscript. WS, BV and KG commented on the first and last draft of the manuscript. All authors have read and approved the final manuscript.

## Supplementary Material

Additional file 1 Two examples of the non-functional and functional approach. Click here for file
