# Supplementary material for: Process evaluation of podiatric treatment of patients with forefoot pain
Source: J Foot Ankle Res. 2013 Aug 7;6:32. doi: 10.1186/1757-1146-6-32 (PMC3750369; doi:10.1186/1757-1146-6-32)
Supplement: Additional file 1 — Two examples of the non-functional and functional approach. [file 1757-1146-6-32-S1.doc]

**Example 1**

Patient 16: Female (age 64)

Pain (≥ 24 months) under MTP 1, 2&3.

**Functional approach**

Aetiology:

Elevated pressure under MTP 1, 2 & 3, Functional hallux limitus due to the calcaneovalgus position, in-toeing during gait. No external rotation ability in hip joint.

Therapeutic goal:

No correction of valgus position calcaneus; it is probably a compensatory position of the inability in the hip for external rotation. Compensate the functional hallux limitus by providing advice to wear a shoe with a toe rocker and that allows for the in-toeing.

**Non-Functional approach**

Aetiology:

Elevated pressure under MTP 1, 2 & 3 and pes transversus.

Therapeutic goal:

Decrease the pressure underneath metatarsal heads by elevating the pressure under the surrounding area’s. Correct calcaneo valgus position.

**Example 2**

Patient 14: Female (age 61)

Pain (≥ 24 months) under MTP 2& 3

**Functional approach**

Aetiology:

Elevated pressure under MTP 2 & 3 due to a hallux rigidus and thus limited ROM in MTP 1. As compensation during gait MTP 2 & 3 are used excessive.

Therapeutic goal:

Realign the gait to use MTP 2 & 3 less by providing advise to wear shoes that contain a toe rocker. The toe rocker shoe might influence the gait line by reducing the amount of dorsal flexion needed in the MTP joints and thus advances the rolling over MTP 1 and less over 2&3.

**Non-Functional approach**

Aetiology:

Elevated pressure under MTP 2 &3.Calcaneo valgus.

Therapeutic goal:

Distribute pressure over bigger area to obtain less peak pressure under MTP 2 and 3 by means of insole with retro capital support. Correct calcaneovalgus position by means of insole with medial support calcaneus.
